# The energy supply security pyramid: A quantitative framework for planning and policy making

**DOI:** 10.1016/j.isci.2025.112407

**Published:** 2025-04-12

**Authors:** Matthias Sulzer, Georgios Mavromatidis, Alejandro Nuñez-Jimenez, Michael Wetter

**Affiliations:** 1 Empa - Swiss Federal Laboratories for Materials Science and Technology, Urban Energy Systems Laboratory, Überlandstrasse 129, Dübendorf, 8600 Zurich, Switzerland; 2 Group for Sustainability and Technology, Swiss Federal Institute of Technology Zurich (ETH Zurich), Weinbergstrasse 56/58, 8092 Zürich, Switzerland; 3 Lawrence Berkeley National Laboratory, Energy Technologies Area, One Cyclotron Road, Berkeley, CA 94720, USA

**Keywords:** Energy policy, Energy management, Energy Modelling

## Abstract

Energy supply security (ESS) is one of the pillars of the Energy Trilemma. Although crucial for the transition to sustainable energy systems, ESS is frequently overlooked in energy system design. This article introduces the ESS Pyramid, a hierarchical framework combining conceptual insights with quantitative metrics to assess supply security. The ESS Pyramid has five levels, including foundational elements, such as Self-Production and Autonomy, and higher-order requirements, such as System Adequacy, Self-Sufficiency, and Autarky. Specific metrics are defined for each level to facilitate quantitative assessments that can inform strategic planning and policy-making. Using Switzerland’s energy transition as a case study, we demonstrate the practical application of the ESS Pyramid and how sustainable transitions can also increase energy supply security. This framework provides policymakers, energy planners, analysts, and researchers with a structured approach that complements the already detailed approaches to Sustainability and Equity, the other two pillars of the Energy Trilemma.

## Highlights

• Energy Supply Security (ESS) is a critical goal for energy policy and system planning
• The ESS Pyramid organizes security indicators into a structure enabling systematic assessment
• Our metrics quantify each ESS Pyramid level to inform strategic planning and policy decisions
• Testing the framework on Switzerland demonstrates its value for renewable energy transitions

## Graphical abstract

**Figure:**
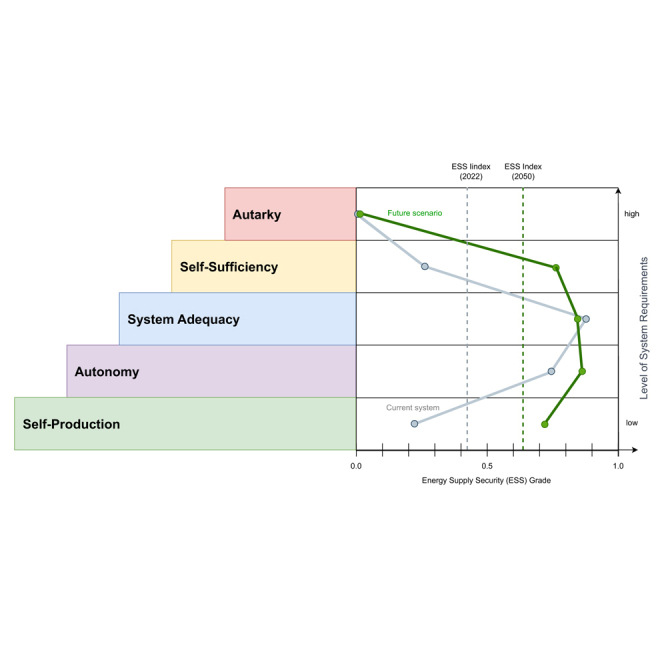

## Introduction

The oil crises of the 1970s revealed how vulnerable global energy systems can be – first in 1973 and again in 1979 – and led to a historical shift in energy priorities. The crises highlighted the importance of energy security for the functioning of advanced economies, making it a primary concern for governments of energy-importing countries. However, this prominence did not last. Oil prices plummeted during the 1980s and remained low until the early 2000s, removing the sense of urgency around energy security. As a result, other priorities filled the vacuum, and energy equity (“Energy equity” is nowadays used to describe the accessibility and affordability of energy supplies across a population, supplanting narrower terms such as “affordability,” “cost,” or “economic efficiency.” Recent literature has expanded this concept beyond cost considerations to address broader equity issues, as shown by Cong et al.^1^) and sustainability – the other two pillars of the so-called Energy Trilemma^2^ (https://www.worldenergy.org/transition-toolkit/world-energy-trilemma-index) – became more salient in energy policy discussions.^3^ Shifts in priorities such as these follow the evolving dynamics of global energy challenges, which are also reflected in the evolution of tools to inform decision-makers such as energy system models.^4^

Recent events, particularly the war in Ukraine, have brought energy security back to the top of governments’ agendas worldwide. Ministers from the G7 nations have underscored the critical necessity of advancing toward not only clean and sustainable but also secure energy systems.^5^

Analyses based on energy system models integrate energy security less frequently than energy equity and sustainability, which are often represented using metrics such as the total cost and CO_2_ emissions of energy supply.^2^^,^^6^^,^^7^

Although well-established methodologies exist for assessing energy equity and sustainability, the literature on energy security encompasses a wide range of perspectives and indicators. This diversity underscores the multifaceted nature of energy security, yet it can also complicate efforts to compare scenarios and inform structured, quantitative decision-making.^8^^,^^9^^,^^10^

Ang et al.^8^ conducted a comprehensive review of 104 studies on energy security, identifying seven major themes: availability, infrastructure, prices, societal effects, environmental impacts, governance, and efficiency. Similarly, Azzuni and Breyer^11^ analyzed 66 definitions of energy security across 15 dimensions, covering technical, social, and geopolitical aspects. In their study, they emphasized the importance of considering non-technical dimensions, such as health, military security, and cultural factors, which are often overlooked in more traditional, technology-centric frameworks. While these reviews provide a structured understanding of what influences energy security, the sheer number of identified dimensions makes it difficult to analyze them and translate the results into actionable strategies.

Moreover, the multiple dimensions are often presented as a flat list, with no cohesive hierarchy indicating how system requirements progress from foundational to more advanced. As a result, policymakers often lack clear guidance on where to begin, making it difficult to address each requirement systematically and prioritize strategic initiatives.

In addition to defining energy security dimensions, many studies have focused on quantifying these dimensions through indicators. Ang et al.^8^ found that 51 of the 104 studies they reviewed utilize indicators to measure energy security dimensions. These indicators provide a way to translate more abstract themes into measurable values, enabling cross-country comparisons and security assessments. However, as the number of indicators increases – exceeding 60 in some studies – the process of operationalizing them in decision-making becomes increasingly complex. Gasser,^9^ in his review of 63 energy security indices, also emphasized that while indicators are helpful in providing quantitative assessments, many lack transparency in how they are selected, weighted, and aggregated, which complicates their use in policymaking and limits their utility for guiding concrete actions. Finally, Månsson et al.^12^ further critiqued existing indicators, noting that many focus on isolated parts of the energy system rather than adopting a comprehensive, system-wide approach. This fragmented approach, along with the complexity of indicators, can complicate the development of cohesive strategies to improve energy security.

Although the literature offers valuable insights into the multifaceted nature of energy security, it also reveals two significant gaps. First, the use of extensive indicator sets and unstructured lists of dimensions complicates prioritization, making it difficult for policymakers and energy planners to develop effective strategies that address the most pressing energy security challenges systematically. Second, many frameworks combine security dimensions with other concerns, such as sustainability (environment and efficiency) and equity (energy prices, societal effects, and governance).^8^ This broad approach, though important, can obscure the distinct and immediate challenges of ensuring energy supply security, particularly in times of crisis. Without a framework that both isolates energy supply security and provides a hierarchy of security dimensions, policymakers may struggle to quantify vulnerabilities adequately and allocate resources effectively.

To address these challenges, we propose a hierarchical framework specifically tailored to Energy Supply Security (ESS). Loosely inspired by Maslow’s hierarchy of needs^13^ (Maslow’s hierarchy serves merely as an inspiration rather than a strict analogy. We recognize that Maslow’s model has been criticized for oversimplification, and we reference it only to illustrate a tiered approach to prioritizing elements of energy security), our framework organizes indicators of ESS into five tiers, ranging from the least demanding at the base (Self-Production) to the most demanding at the apex (Autarky). Each tier reflects an increasingly stringent level of requirements for addressing energy security, offering policymakers, planners, and analysts a clear structure for systematically evaluating and prioritizing ESS objectives.

The framework also includes corresponding metrics for each tier, offering a flexible approach that can be adapted to various modeling contexts and needs. By systematically measuring each dimension, planners can weigh trade-offs, identify the most effective measures for strengthening energy security, and determine which strategies best suit their unique contexts. This quantitative approach can be integrated into existing optimization models that already incorporate aspects such as costs and emissions to enable an even more comprehensive evaluation of energy systems.^14^

Our work builds on and expands the existing body of research with a hierarchical quantitative framework that consolidates five dimensions of ESS. Through this structured approach, we aim to complement—rather than replace—the extensive research already available, thereby enabling policymakers and analysts to systematically assess energy security, particularly in the context of the sustainable energy transition.

While our approach is rooted in the overarching concept of the Energy Trilemma, this article focuses exclusively on energy security as a component distinct from energy equity and sustainability.^7^ We acknowledge that a comprehensive energy system analysis must ultimately address all three dimensions and their interdependencies. However, we deliberately isolate energy security in our framework to provide a clearer basis for examining trade-offs among security, economics, and sustainability. This approach aligns with earlier observations by Ang et al.,^8^ who note that *“although there is due consideration for the economic and environmental dimensions of energy security […], a formal treatment in the energy security literature of the concept of energy trilemma in a comprehensive and rigorous manner is lacking.”*

The rest of the article is structured as follows: Section results first introduces the Energy Supply Security Pyramid (ESS Pyramid), defining the different levels of ESS. Then, in a demonstrative case, it applies the concept of the ESS Pyramid and the corresponding quantitative metrics defined in the STAR Methods section to investigate Switzerland’s current and future energy system. Finally, the Section discussion discusses our study’s limitations, provides concluding remarks, and discusses paths for refining the analytical framework.

## Results

### The Energy Supply Security Pyramid

ESS can be understood as the fundamental need of energy systems to match different energy supplies (domestic vs. imported) to different energy demands (essential vs. non-essential) across various time frames (every moment vs. over a period). Domestic energy supplies are produced within the energy system’s boundaries (e.g., within a country’s borders), while imported supplies are brought in from elsewhere. Essential energy demands relate to the fundamental infrastructure required for delivering essential services, such as hospitals and water supply.^8^^,^^11^ Non-essential demands include all other energy needs. (In advanced economies, nearly all energy demands are considered essential due to the interdependence of sectors and activities, though distinct critical infrastructures often rely on stand-alone backup plants. In our Swiss case study (see Section case study: Energy Supply Security and the case of Switzerland), we treat all demands as essential. Nevertheless, some uses (e.g., sporting events or recreational activities) are not strictly essential. In developing economies, essential needs may be limited to specific critical services, reflecting different social and geopolitical conditions.) The time frames for matching supply and demand can be analyzed either as an aggregated balance over the analyzed period (*T*), typically one year, or, more technically demanding, for any specific moment (dt), typically in hours, quarter hours, or even sub-minutes, throughout the analyzed period.

**Figure 1:**
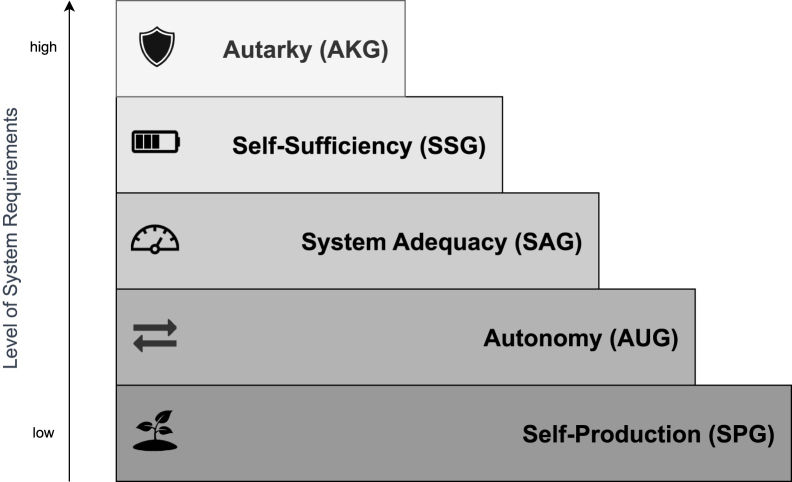
The Energy Supply Security Pyramid

The way various energy supplies meet energy demands can be characterized by the three key aspects introduced above (Table 1) and organized into five hierarchical levels - Self-Production, Autonomy, System Adequacy, Self-Sufficiency, and Autarky - as illustrated in Figure 1. These levels represent progressively more stringent requirements for energy system reliability and control.

**Table 1:** Energy supply aspects are hierarchically ordered according to the energy system’s requirements of demands met, supplies used, and temporal matching

Levels of ESS	Energy System Aspects					
	Demands that can be met		Supplies that are used		Supplies match demands	
	Essential	Non-essential	Domestic	Imported	Every moment	Over the analyzed period
*Autarky*	✓	✓	✓		✓	✓
*Self-Sufficiency*	✓	✓	✓	✓^b^	✓	✓
*System Adequacy*	✓	✓	✓	✓	✓	
*Autonomy*	✓	✓	✓^a^	✓		✓
*Self-Production*	✓		✓			✓

a Domestic supplies are considered to have the highest level of autonomy, as the nation could exert full control over them.

b Minimizing imports during the analyzed period.

Lower levels of the ESS Pyramid examine whether a country, region, or system can produce or import enough energy over a given period (e.g., annually), considering its mix of energy carriers. At the strategic policy level, such indicators are commonly used to assess an energy system’s overall capability. However, an aggregated evaluation does not guarantee a continuous, uninterrupted supply at every moment in time. The higher levels of the ESS Pyramid address these reliability concerns more explicitly, examining infrastructure adequacy, temporal demand fluctuations, and the capacity to prevent or mitigate supply interruptions. To provide a comprehensive energy security analysis, it is crucial to analyze all levels of the pyramid in tandem.

Each level is defined as follows (see Table 1).

(1) Self-Production: The ability to meet essential energy demand, at least in the aggregate, using domestic supplies over the analyzed period. Failure to meet essential demand indicates insecurity.
(2) Autonomy: The ability to meet both essential and non-essential demands, in the aggregate, using a combination of domestic and imported supplies over the analyzed period.
(3) System Adequacy: The ability to meet all demands (essential and non-essential) at every moment in time, not just in aggregate, using domestic and imported supplies.
(4) Self-Sufficiency: The ability to meet all energy demands at every moment using only domestic resources during specified periods while minimizing reliance on imports over the entire analyzed period.
(5) Autarky: The ability to meet all energy demands exclusively with domestic resources at every moment and over the entire analyzed period.

Achieving lower levels often contributes to higher-level objectives^15^; for example, increased domestic supply enhances both Self-Production and Self-Sufficiency. While this framework offers a valuable initial structure, it is not presented as the only valid approach.

When viewed collectively, high ESS is characterized by consistently fulfilling both essential and non-essential demands, instantaneously and in the aggregate, with only a moderate reliance on imports.

#### Self-Production

Self-Production, the foundational level in the ESS Pyramid, represents an energy system’s ability to meet its essential energy demands, at least in the aggregate, with domestic supplies over the analyzed period. This base level of energy security allows the energy system to support essential societal functions such as safety, health, and food and water supply.^16^

At this level of ESS, the energy system relies on domestic energy supplies. These may include stocks such as coal, oil, and gas reserves, as well as solar, wind, hydro, and biomass resources, which are used to balance essential demands within the analyzed time horizon.

Increasing the energy system’s requirements beyond Self-Production, the energy system has to meet essential and non-essential demands in the analyzed period by either expanding domestic resources or incorporating imported supplies.

#### Autonomy

Autonomy, the second level of the ESS Pyramid, represents an energy system’s ability to meet essential and non-essential energy demands, at least in the aggregate, using a combination of domestic and imported supplies over the analyzed period. Energy security at this level can be enhanced through various more robust and secure imports, as well as by building strategic reserves that are readily accessible during unexpected events.

At this level of ESS, the energy system uses domestic and imported energy supplies. By establishing agreements, partnerships, and market integration with other countries, the system becomes less vulnerable to disruptions. This approach enhances the system’s ability to guarantee the long-term matching of supply and demand, thereby providing a greater level of security.^17^^,^^18^^,^^19^

Increasing the energy system’s requirements beyond Autonomy, the energy system has to meet essential and non-essential energy demands consistently at every moment within the analyzed period. A system may achieve high autonomy; however, if it cannot sufficiently distribute energy to consumers at every moment, it will fall short of attaining high system adequacy, as outlined in the next level.

#### System Adequacy

System adequacy, the third level of the ESS Pyramid, represents an energy system’s ability to meet both essential and non-essential energy demands at every moment over the analyzed time period using a combination of domestic and imported supplies. This level ensures that the system can maintain an uninterruptible supply by adequately meeting all energy demands at any given moment, hence contributing to economic stability and social welfare.^10^

At this level of ESS, the energy system ensures supply stability and minimizes the risk of disruptions by maintaining adequate backup capacity through energy stocks. Meeting all energy demands at every moment over the analyzed time period is a prerequisite for the energy system’s ability to cope with system dynamics. Providing flexibility is essential for System Adequacy, as is offering reserve capacity, either domestically or through import routes.

Increasing the energy system’s requirements beyond System Adequacy, the energy system has to minimize imported supplies over the analyzed time period by maximizing the self-consumption of domestic resources.

#### Self-Sufficiency

Self-Sufficiency, the fourth level of the ESS Pyramid, represents an energy system’s ability to meet its essential and non-essential energy demands at every moment over specific periods using only domestic resources. This level of energy security allows the energy system to function without imported supplies over defined time periods, ranging from several days to even months.

At this level of ESS, the energy system can balance supply and demand at every moment, relying solely on domestic energy supplies during specific periods. Outside of these “self-sufficient” periods, imports are still utilized. The longer the “self-sufficient” periods last, the fewer imports are needed in the long term. By investing in domestic energy resources and storage systems, the system can significantly extend the duration during which no imports are necessary.

Increasing the energy system’s requirements beyond Self-Sufficiency, the energy system has to meet both essential and non-essential energy demands at every moment and over the analyzed time period using only domestic resources.

#### Autarky

Autarky, the fifth and highest level of the ESS Pyramid, represents an energy system’s ability to meet its essential and non-essential energy demands using only domestic supplies at every moment over the whole analyzed period. This level of energy security allows the energy system to rely on its own resources indefinitely, effectively becoming an energy island.^20^

An autarkic energy system exclusively uses domestic energy supplies. This requires sufficient domestic supplies to address energy demand across daily and seasonal variations and through shocks and disruptions. All reserves and backup plants are part of the domestic resources. By achieving Autarky, the system fulfills the most advanced requirements and hence achieves the highest level of ESS.

After reaching Autarky, further increasing the energy system’s requirements is not possible. However, there are several ways to continue improving the ESS, as we explain in the next section.

#### Cross-cutting measures to enhance energy supply security

Several important aspects of how an energy system matches supply and demand can increase its security within each ESS level. Among the most relevant are the number and variety of supplies (diversity vs. concentration), the durability of supplies (renewable vs. finite), the scale and types of storage options, and the technological sovereignty.

The concentration of energy supplies can reduce an energy system’s ability to match supply and demand when faced with disruptions.^21^ Within each level of ESS, a system with more diverse and distributed energy supplies will be more secure, due to the increased resilience against supply disruptions and demand shocks.^22^ Agreements and partnerships with neighboring systems and cross-border connections with energy networks, such as electricity and gas grids, can increase the diversity of imported supplies and enhance the security of energy systems that use imports.

Finite energy supplies, whether domestic or imported, risk depletion over time. The ability to match the supply and demand of an energy system based on finite energy supplies, such as oil, coal, and gas, relies on the abundance and accessibility of these resources in both the short and long term. Therefore, increasing the proportion of renewable energy supplies can improve the energy security of the system at every level of ESS.

Storage can enhance an energy system’s security at every level of the ESS pyramid by mitigating the need to match supply and demand instantaneously. Technologies such as batteries and hydropower reservoirs can help match supply and demand in the short term (e.g., minutes, hours, days), while strategic storage options such as oil and gas reserves, as well as emerging solutions such as hydrogen and synfuels, can address more prolonged disruptions (e.g., across weeks, months, seasons). When combined with fundamental changes in the energy system, such as expanding domestic supplies, the ability of storage options to absorb excess supply and use it during periods of peak demand can facilitate the move toward higher ESS levels (e.g., from Self-Production to Self-Sufficiency).

Finally, access to the necessary technology to build and operate energy systems is a critical aspect of ESS at all levels. Technological sovereignty involves ensuring that the country has access to key technologies and components required for its energy system. This control can be achieved by fostering domestic innovation, securing intellectual property rights, and developing local manufacturing capabilities. It also involves diversifying supply chains to reduce dependency on any single foreign supplier, which can mitigate risks associated with geopolitical tensions or trade disruptions. Building capacity in the workforce is equally important. This includes research, education, and training programs to develop the technical skills and innovations required to design, build, and maintain advanced energy systems.

### Case study: Energy supply security and the case of Switzerland

To illustrate the application of the ESS Pyramid and its corresponding metrics, described under the method details in the STAR Methods section, we examine Switzerland’s current situation (Section the historical and current energy supply) and a future scenario (Section the future of the swiss energy supply), exploring the nation’s historical reliance on imports and its transitional efforts toward a more resilient and sustainable energy system. The quantitative metrics used for the analysis are defined in the method details of the STAR Methods section.

#### The historical and current energy supply

**Figure 2:**
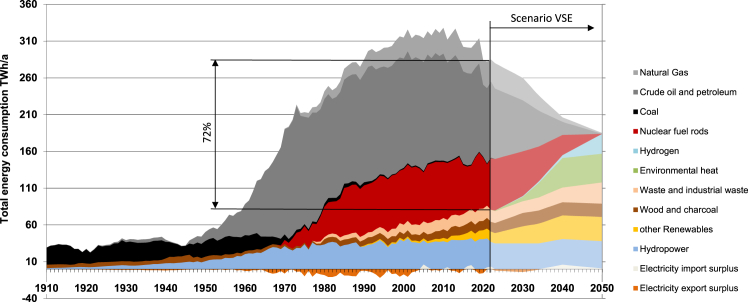
Gross energy consumption of Switzerland since 1910, Schweizerische Gesamtenergiestatistik 2022, SFOE^25^ The Scenario VSE^26^ depicts the transformation of the energy system, envisioning the scenario with complete integration into the EU’s electricity and hydrogen networks, along with a strong societal acceptance of new technologies.

Switzerland’s energy landscape has been characterized by over a century of heavy reliance on energy imports, accounting for more than 70% of the gross energy consumption (281 TWh/a), as shown in Figure 2. Historically, these imports have been dominated by coal, while currently, fossil fuels (crude oil and natural gas) account for the majority of imports. The nation has maintained a relatively balanced production-consumption equation for electricity but faces challenges in broader energy Self-Production, the foundational level of the ESS pyramid. Following the decision to phase out nuclear power until 2034,^23^ the Autonomy level of imports declines, which undermines the future overall supply security. The expansion of renewable energy technologies such as photovoltaic (PV), wind, biomass, and hydroelectric power has been modest over the last two decades, positioning Switzerland at the lower end of the scale in Europe for the addition of new energy production capacity per capita.^24^

**Figure 3:**
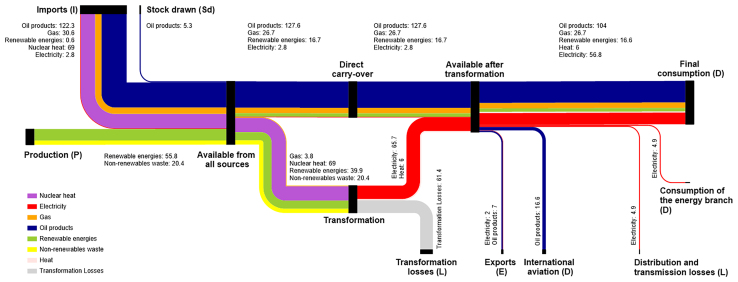
Energy flows in TWh/a of Switzerland 2022 according to the Schweizerische Gesamtenergiestatistik 2022, Swiss Federal Office of Energy (SFOE)

The analysis of the current state of ESS draws information from the “Gesamtenergiestatisik 2022” dataset published by the Swiss Federal Office of Energy.^25^ The energy flows for the analyzed year are illustrated in Figure 3, the gross energy consumption is shown as *Available from all sources* (total of 306.9 TWh), including international aviation (16.6 TWh/a), exports (9 TWh/a). The hourly time series of these energy flows are generated by the model-based approach developed in the “VSE Energiezukunft 2050” project.^26^ Based on these time series, the ESS Grades and the overall ESSI are calculated, as shown in Table 2.

**Table 2:** Comparison of energy supply security Grads and index of the Swiss current and future energy systems

Level of ESS	Current Energy System (2022)					Future Energy System (2050)				
	w	a	n	c	Grades	w	a	n	c	Grades
AKG	0.2				0.00	0.2				0.00
SSG	0.2				0.27	0.2				0.77
SAG	0.2				0.86	0.2				0.84
Crude oil				0.80					0.80	
Natural gas				0.80					0.80	
Nuclear fuel				1.00						
Electricity				0.94					0.78	
Hydrogen									0.80	
Environ. heat				1.00					1.00	
Wood				1.00					1.00	
AUG	0.2				0.74	0.2				0.87
Crude oil		1.00	1.00				1.00	1.00		
Natural gas		0.50	1.00				0.50	1.00		
Nuclear fuel		0.30	1.00							
Electricity		0.70	1.00				0.70	1.00		
Hydrogen							0.50	1.00		
SPG	0.2				0.21	0.2				0.72

**ESSI**					**0.42**					**0.64**

The Self-Production Grade (SPG) of the current energy system is 0.21. In our case study, the essential demand *D* for Switzerland is regarded as the whole domestic demand, excluding any export requirements *E*

The Autonomy Grade (AUG) is equal to 0.74. This value is derived from autonomy factors *a* for each energy carrier, determined by the number of import routes. Specifically, each carrier’s a-factor reflects its level of import accessibility: 0 if fully dependent on single import routes and suppliers, 0.3 for nuclear fuels (4 suppliers), 0.5 for natural gas (11 feed-in points), 0.7 for electricity (41 connection points to the EU grid), and 1.0 for crude oil (any supply points). The threshold for strategic reserves is set to $n_{i}=1.00$, indicating that maximum autonomy can be achieved with a storage capacity sufficient to meet at least one year of the corresponding demand. In 2023, Switzerland maintained strategic reserves sufficient for approximately 3–4.5 months of electricity and fossil fuels and about one year’s supply of nuclear fuel rods.^27^

The System Adequacy Grades (SAG) is 0.86, with the capacity factor *c* for each energy carrier estimated by us as follows: 0 if no dispatchable capacity is available, 0.5 if various and combined but stochastic-based capacities are available, typically represented by photovoltaic and wind power plants, 0.8 if some technologies have a backup, or 1.0 if *N*-1 criterion^28^ and full control of dispatching applies for any technology, as seen in the interconnected systems of hydro-power plants. The capacity factor, calculated as a weighted average of the electricity production mix, is 0.94, based on the weighted proportions of the resources used.

The corresponding Self-Sufficiency Grade (SSG) is 0.27, and the Autarky Grade (AKG) is 0, as Switzerland depends on imports to meet its national energy demands.

To compute the overall ESS Index, we adopt an equal weighting approach for additive aggregation, thereby assigning the same importance to each of the five ESS Grades. This method aligns with recommendations in the literature and ensures transparency by treating each dimension as equally crucial for assessing energy supply security.

Given equal weights, evaluating (1) yields an ESSI for Switzerland in 2022 of 0.42. This index should be analyzed either in comparison to other energy systems with similar contexts and boundaries, such as other countries, or over time to observe trends. We will apply the latter approach and calculate the ESSI for a Swiss energy supply scenario in 2050.

Comparison of our ESSI with other indices, such as the World Energy Council’s (WEC) Energy Index,^29^ offers valuable insights. While the WEC assigned Switzerland an energy security score of 0.64 in 2023, direct numerical comparison with our ESSI is precluded by differences in assessment dimensions. The WEC index incorporates factors such as diversity of total energy supply, import independence, diversity of suppliers, diversity of electricity generation, storage, system stability and recovery capacity—some of which are reflected in our SPG, AUG, and SAG metrics. However, our ESS Pyramid uniquely incorporates higher-order requirements (Self-Sufficiency, SSG and Autarky, AKG), crucial for evaluating renewable energy systems. An equal-weighted ESSI calculation considering only SPG (0.21), AUG (0.86), and SAG (0.74), see Table 2, yields a value of 0.60. WEC reported an Energy Security Score of 0.645 in 2023 for Switzerland. Although not directly comparable, this reduced-scope ESSI provides a qualitative benchmark, contextualizing our findings within the broader landscape of energy security assessments.

Furthermore, Switzerland’s ranking in the WEC Trilemma indices (https://trilemma.worldenergy.org/) supports our findings regarding its relatively modest energy supply security: while ranking 3rd overall across all Energy Trilemma dimensions, its energy security performance placed it 33rd globally.

#### The future of the Swiss energy supply

Marti et al.^26^ present a cost-effective, net-zero carbon emission scenario for Switzerland’s energy system by 2050. This scenario includes integration with the EU energy infrastructure, phasing out nuclear power plants (−23 TWh/a), and significantly expanding renewable (+32 TWh/a) and hydrogen-based electricity production (+13 TWh/a). Transitioning from fossil fuel boilers to heat pumps enables the substantial utilization of local environmental heat (+34 TWh/a) and industrial waste heat (+11 TWh/a) (see Figure 2).

**Figure 4:**
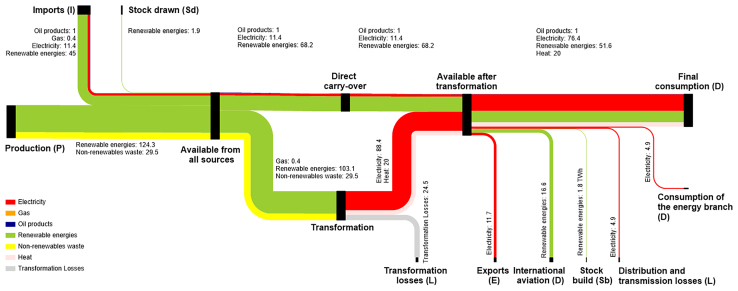
Energy flows in TWh/a of Switzerland 2050 according to the VSE Energiezukunft 2050, “integriert-offensiv” secenario

The study suggests that Switzerland can increase its Self-Production to cover two-thirds of the projected total energy consumption of 185 TWh/a (Figure 2). When including additional demands such as international aviation (16.6 TWh/a) and exports (11.7 TWh/a), the total energy requirement —referred to as *Available from all sources* in Figure 4—amounts to 213 TWh/a by 2050. The reduction in domestic energy consumption from 281 TWh/a to 185 TWh/a. Figure 2 demonstrates that efficiency gains are primarily attributable to electrification of the heating and mobility sectors, coupled with improvements in buildings and appliances. However, electrification will increase electricity final consumption from 57 TWh/a to 76 TWh/a by 2050 (see Figures 3 and 4).

Analyzing the five ESS dimensions and overall ESSI for 2050 identifies both Switzerland’s projected security trends and specific areas requiring attention to maintain high supply security. The following calculation exemplifies the assessment for a single scenario, demonstrating how our framework can be applied to evaluate future ESS. These metrics can also be directly integrated into system design algorithms to generate supply scenarios themselves.^9^

The future ESS Grades and Index are presented in Table 2. The parameters *a* and *n* were applied consistently with the ones used for the ESS calculation of the current energy system. The capacity factor *c* is adapted for the new mix of electrical energy carriers to 0.78, as they will be more stochastically dominant in 2050. Overall, the resulting ESSI for the future scenario will be 0.64 in 2050.

#### The impact on ESS over time

**Figure 5:**
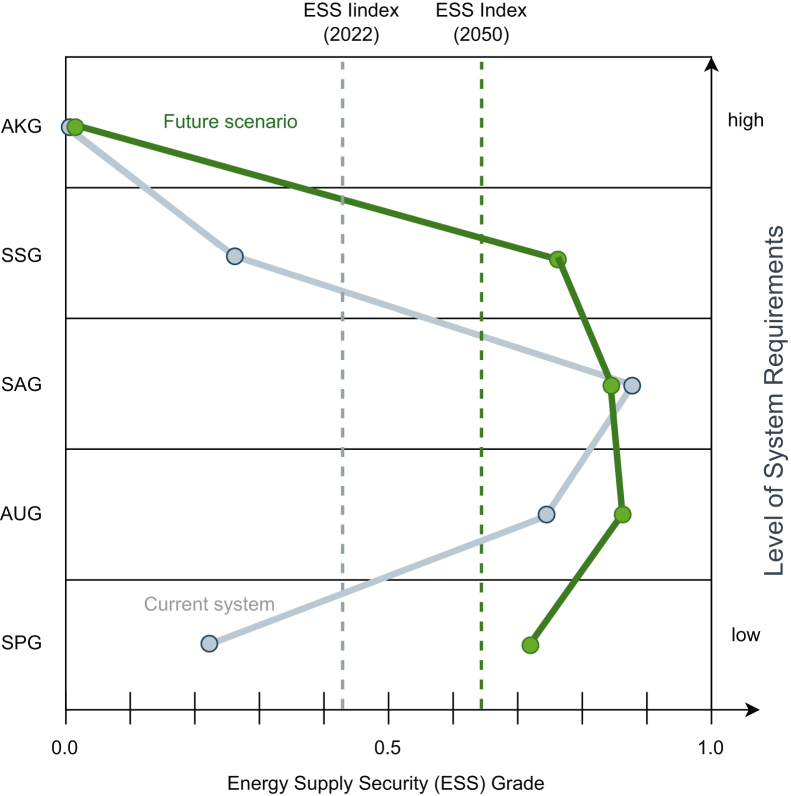
Comparison of the current and future Energy Supply Security Index (ESSI), illustrating the contributions from each of the five levels of the ESS Pyramid

Overall, Switzerland’s ESSI in the analyzed scenario improves from 0.42 to 0.64 as it transitions toward a renewable energy system with more domestic production, as shown in Figure 5. However, not all elements of energy supply security move in the same direction. Comparing the indicators of each ESS level of the current and future systems helps identify the drivers of change and demonstrate the usefulness of the ESS Pyramid.

##### Self-Production (SPG)

The SPG of the Swiss energy system is projected to improve substantially from 0.21 in the current system to 0.72 in the future system. This significant enhancement reflects the country’s ambitious targets to increase domestic renewable energy production. However, despite these improvements, Switzerland will continue to rely on energy imports, renewable electricity, and hydrogen to maintain competitive energy prices.^26^

##### Autonomy (AUG)

The AUG increases slightly from 0.74 in the current system to 0.87 in the future system. This improvement is primarily due to a significant reduction in energy imports as Switzerland shifts toward greater self-production from domestic renewable sources. Currently, Switzerland relies on imports of fossil fuels (a=1.0) and nuclear fuel rods (ϑ=1.0), which offer high import flexibility and autonomy through diversified global markets and substantial strategic reserves. In the future, phasing out nuclear power plants and reducing fossil fuel imports lead to increased reliance on hydrogen (a=0.5) and electricity (a=0.7) imports. Despite potential reductions in autonomy due to the nature of hydrogen and electricity imports, the overall decrease in import volume compensates for this, resulting in a net improvement in AUG. Engaging in international energy markets and fostering robust partnerships can further enhance energy autonomy, e.g., increasing the *a* factor by diversifying sources and technologies. Careful management of these international relationships can moderate dependency on external entities while maintaining sovereignty.^30^^,^^31^

##### System adequacy

The SAG shows a slight decrease from 0.86 in the current system to 0.84 in the future system. This minor reduction is primarily due to the increased integration of more intermittent renewable energy sources, such as PV and wind power, which have lower capacity factors. Specifically, the capacity factor *c* for electricity has decreased from 0.94 to 0.78, reflecting the intermittency of these renewable sources. The higher variability of their production is partly offset by the deployment of hydrogen power plants. Such plants can provide dispatchable power generation when renewable output is low, thereby helping to stabilize the overall energy system. Flexibility within the energy system is crucial for System Adequacy.^32^ Additionally, demand-side policies such as energy efficiency measures and demand response programs can enhance System Adequacy. By reducing overall demand and aligning consumption with resource availability, these policies effectively improve the utilization of available capacity, which can be reflected by higher capacity factors *c*.

##### Self-Sufficiency (SSG)

The SSG sees a massive increase from 0.27 in the current to 0.77 in the future energy system. This substantial improvement is made possible by the transformation of the energy system, particularly through the electrification of the heating and mobility sectors. Electrification enables greater utilization of domestic energy infrastructure, including renewable energy production, advanced storage solutions, and digital services. The integration of smart grid technologies and energy management systems, e.g., demand-side management (DSM), enhances the efficiency and flexibility of the energy supply, further bolstering Self-Sufficiency.^33^^,^^34^

##### Autarky

The AKG remains at 0.0, indicating that Switzerland will not achieve complete energy autarky by 2050. Despite significant increases in domestic renewable energy production and reduced energy imports, the country continues to rely, at least partly, on imported resources. The VSE case study shows that attaining full energy autarky may not be economically viable and could potentially undermine Switzerland’s competitiveness by resulting in higher energy costs.^26^ However, achieving autarky for essential services—such as hospitals, water supply, telecommunications, and military operations—can be an effective strategy to protect critical infrastructure during extreme events. By integrating a dedicated backup plant into the national energy system, Switzerland can enhance its resilience to blackouts while upholding economic efficiency and sustainability. This approach balances the advantages of international energy cooperation with the need to safeguard vital services.

The analysis of indicators at each level of the ESS Pyramid highlights the key drivers behind the potential improvement in Switzerland’s ESSI. A major factor contributing to this improvement is the substantial reduction in the total volume of energy imports due to the increased domestic production of renewable energy and the electrification of heating and mobility. Although the reliance on imported energy carriers such as hydrogen and renewable electricity introduces reduced autonomy—owing to dependence on specific supply infrastructures—the significant decrease in overall import volumes more than compensates for this effect. Consequently, the transition to a greener energy system enhances Switzerland’s ESSI, reflecting an overall improvement in the nation’s energy supply security. Interestingly, Kim et al. find similar outcomes in their recent study about energy security and the green transition.^21^

### Limitations

While the ESS Pyramid framework presents a useful tool for research on energy security, it has some limitations.

First, data for calculating all metrics and parameters may not always be available. While parameters such as the autonomy factor *a* and the reserve factor *n* can be quantified using the REES framework^35^ or derived from governmental reports, other parameters such as the capacity factor *c* used in calculating the SAG grade may often require expert assessments. Additionally, accurate assessment of System Adequacy requires simulation using dynamic network models, given the complexity and interdependencies of modern energy systems.

Second, aggregation into a single index number requires carefully considering the impact of weighting. We chose equal weights in our analysis, but this approach has trade-offs. Different weighting schemes can tailor metrics to a nation’s specific energy circumstances,^36^ but may introduce bias. Transparency in weight selection is, therefore, essential for accurate ESSI representation.

Varying weights also limit cross-country ESSI comparability of ESSI scores between countries, as countries with identical ESSI values may have fundamentally different energy security profiles. For meaningful comparisons, either standardized weights must be adopted or the weights used must be transparently reported. For instance, Japan, with limited space and natural resources, might prioritize Autonomy due to its heavy reliance on energy imports, while resource-rich Australia might emphasize Self-Production and Self-Sufficiency. While this tailored approach provides contextually relevant measurements, it reinforces the need for transparent weight reporting to minimize bias and maximize comparative value.

## Discussion

This research presents the Energy Supply Security (ESS) Pyramid, a comprehensive and hierarchical framework that combines conceptual insights with quantitative metrics to offer a clear and measurable approach to assessing energy supply security. The ESS Pyramid highlights foundational elements such as Self-Production and Autonomy while also addressing higher-order needs such as System Adequacy, Self-Sufficiency, and Autarky. By evaluating each level, the framework considers both technical and non-technical dimensions of energy security. The hierarchical structure of system requirements and tier-specific indicators enables stakeholders to effectively design and evaluate the energy system’s supply security.

Applying the ESS Pyramid to Switzerland’s energy system reveals both strengths and areas for improvement, particularly as the country transitions toward renewable energy sources. Our analysis indicates that Switzerland has the potential to substantially improve its energy supply security by expanding Self-Production, such as PV and wind energy. However, to mitigate the intermittent nature of these renewables, deploying flexible hydrogen power plants is necessary to maintain a high degree of System Adequacy. Additionally, non-technical measures, such as the European energy market integration and establishing robust supply treaties, can further enhance the ESSI.

A foundational prerequisite for basic energy supply security is ensuring sufficient production and imports to meet demand within a specified time frame. These lower-level requirements are often attainable in developing nations. However, transitioning to a more reliable energy infrastructure calls for higher-level requirements, such as System Adequacy and Self-Sufficiency. Implementing these measures typically involves substantial investments and technological innovation, which in turn help mitigate supply disruptions and promote sustainable economic growth. As nations progress along this pathway, they strengthen their energy resilience by consistently meeting demand and fostering economic prosperity.

By isolating security in a ceteris paribus exercise, designers, policymakers, and analysts can more accurately quantify and compare security outcomes before reintroducing economic and sustainability constraints, thus clarifying the trade-offs among the three dimensions of the Energy Trilemma. The ESS Pyramid is designed to complement existing tools focused on cost or sustainability factors, allowing for systematic assessments of how these elements can be balanced.

## Outlook

Future research can extend the application of the ESS Pyramid to other countries, enabling comparative analyses and refinement of the framework. Integrating the ESS metrics into energy system modeling and optimization tools will facilitate the development of energy strategies that holistically address security, sustainability, and economic considerations.

There is also a need to develop additional, nuanced metrics tailored to specific system needs at different scales. Enhancing the ESS Pyramid to analyze subsystems within the broader energy system—such as electricity grids, hydrogen infrastructure, renewable energy integration, and critical infrastructure resilience—will provide a more granular understanding of energy security. Developing specific metrics for these subsystems will enable more targeted and actionable assessments.

Future research should focus on refining the quantification of the capacity factor *c* to establish a more direct, standardized calculation that does not depend extensively on network modeling. Achieving a methodology that can be seamlessly integrated into the SAG grading framework is crucial for both holistic system design algorithms and rigorous numerical evaluations of energy systems.

Addressing cyber vulnerabilities and enhancing system agility are critical components of modern energy security. Future research should focus on developing robust metrics that gauge cybersecurity, including the resilience of infrastructure against cyberattacks and the effectiveness of response and recovery mechanisms. Additionally, metrics for system agility would capture the flexibility and adaptability of energy systems in the face of extreme or unforeseen events. Building on these considerations, future work should explicitly integrate cybersecurity indicators, recognizing that protecting digital infrastructure is vital for maintaining a reliable energy supply. By incorporating vulnerability assessments into the existing ESS metrics, future work should reflect the interplay between physical and digital threats, offering a more thorough and adaptive approach to safeguarding energy systems.

## Resource availability

### Lead contact

Requests for further information and resources should be directed to and will be fulfilled by the lead contact, Matthias Sulzer (Matthias.Sulzer@empa.ch).

### Materials availability

This study did not generate new unique reagents.

### Data and code availability

• Data: All data reported in this article will be shared by the lead contact upon request.
• Code: The data from the case study and the Python code used for calculating the ESS metrics are available at https://github.com/lbl-srg/energy-supply-security-pyramid. This allows replicating the presented results.
• All other items: Any additional information required to reanalyze the data reported in this article is available from the lead contact upon request.

## Acknowledgments

This research was supported by the Assistant Secretary for Energy Efficiency and Renewable Energy, Office of Building Technologies of the U.S. Department of Energy, under Contract No. DE-AC02-05CH11231, the 10.13039/501100005380Swiss Federal Office of Energy as part of the SWEET consortium PATHFNDR (Nr. SI/502259-01), and GOES-CH, Optimierte Energiesysteme mit Geothermie (Nr. SI/502485-01). We extend our gratitude to Martin Rüdisüli from the Verband Schweizerischer Elektrizitätsunternehmen (VSE) for providing the model data of the Energiezukunft 2050 study. Additionally, we would like to thank Prof. Dr. David N. Bresch for his valuable comments on the first draft of this article, Amer Gharib of the World Energy Council for providing the Swiss Energy Security Indicators and Dr. Lucas Miehé and Emily Lennon for reviewing an early version of the article.

## Author contributions

Matthias Sulzer: conceptualization; methodology; software; validation; formal analysis; investigation; data curation; writing – original; writing – review and editing preparation; visualization; and project administration. Georgios Mavromatidis: conceptualization; methodology; validation; formal analysis; investigation; writing – original; writing – review and editing preparation; and visualization. Alejandro Nuñez-Jimenez: conceptualization; methodology; validation; formal analysis; investigation; writing – original; writing – review and editing preparation; and visualization. Michael Wetter: conceptualization; methodology; software; validation; formal analysis; investigation; data curation; writing – original; writing – review and editing preparation; and visualization.

## Declarations of interests

We hereby declare that the content of this document was conceptualized and originated by the study’s main author and co-authors. While we utilized tools such as ChatGPT, Deepl, and Grammarly to improve the writing and ensure grammatical accuracy, these tools were not employed to generate any of the core content or ideas presented herein.

## STAR★Methods

### Key resources table

**Table:** 

REAGENT or RESOURCE	SOURCE	IDENTIFIER
**Deposited data**		

Swiss energy statistics 2022	Swiss Federal Office of Energy (SFOE)	https://www.bfe.admin.ch/bfe/en/home/supply/statistics-and-geodata/energy-statistics/overall-energy-statistics.html/
Hourly time series for Swiss energy flows - Reference scenario	VSE Energiezukunft 2050 project	Marti et al.^26^; https://github.com/lbl-srg/energy-supply-security-pyramid
Hourly time series for Swiss energy flows - Scenario 2050	VSE Energiezukunft 2050 project	Marti et al.^26^; https://github.com/lbl-srg/energy-supply-security-pyramid

**Software and algorithms**		

Python 3.8.10	Python Software Foundation	https://www.python.org/
Energy Supply Security metrics and Index calculation	This paper	method details

### Method details

#### Metrics for Energy Supply Security Index

The tiers of the ESS Pyramid presented in Section results provide the conceptual foundation of our framework in support of energy system planners, policymakers, and researchers tasked with analyzing energy systems on various scales - from buildings to nations. In this section, the ESS tiers are complemented by quantitative metrics that can provide further insights into their efforts (In-depth analysis for subsystems might require adapted or refined metrics. For example, power quality analysis in electrical grids requires additional metrics to investigate frequency or voltage stability, while analysis of thermal grids necessitates temperature threshold alignment. To address in-depth supply security analysis, a platform-based design provides a systemic framework^37^). While the conceptual definitions of the levels encompass multiple dimensions—technical ones that can be calculated based on measurements or simulation results, and non-technical ones that need to be estimated by experts—the quantitative metrics capture the essence of their key dimensions in a formal way to ensure they can be readily integrated into model-based energy system analyses.^14^ In their review, Ang et al.^8^ highlight a high degree of subjectivity in constructing energy security indices, particularly regarding the choice of accounting frameworks and the assignment of weights. They also note that equal weighting and additive aggregation are the most frequently employed methods. Based on these findings, we recommend using an equally weighted average to compute the overall Energy Supply Security Index (ESSI).

Nevertheless, if a particular region or context necessitates placing greater emphasis on one dimension over another—thus capturing the unique circumstances of an energy system’s provision—decision-makers can adjust the weighting scheme accordingly.^36^ Alternatively, they may employ more sophisticated methods for computing an overall index, such as a weighted geometric mean, harmonic mean, or multi-criteria decision analysis (MCDA).^38^

It is important to note that the selection of weights and the accounting method may introduce bias; therefore, transparency is essential to understand how the ESSI is represented. Moreover, using different weights can limit the comparability of ESSI scores between systems or countries.

By aggregating the five ESS grades, the ESSI becomes an effective metric for quantifying an energy system’s security—particularly in the realms of energy system design^37^ and optimization.^39^^,^^40^^,^^41^

Moreover, this conceptual framework can be seamlessly integrated into energy system models, allowing planners to evaluate alternative designs based on the ESSI.

In contrast, relying solely on the aggregated ESSI may obscure important nuances. By comparing the individual metrics at each level of the ESS Pyramid, analysts can gain deeper insights—valuable for comprehensive energy system evaluation. Assessing each dimension individually also ensures a uniform evaluation of components without strictly invoking the hierarchical structure.

The overall Energy Supply Security Index (ESSI) is calculated as a weighting and additive aggregation of the grades assigned to each level of the ESS Pyramid:

(Equation 1) $$ESSI=\frac{w_{1}\;SPG+w_{2}\;AUG+w_{3}\;SAG+w_{4}\;SSG+w_{5}\;AKG}{w_{1}+w_{2}+w_{3}+w_{4}+w_{5}}\text{,}$$

where SPG is the Self-Production Grade, AUG is the Autonomy Grade, SAG is the System Adequacy Grade, SSG is the Self-Sufficiency Grade, AKG is the Autarky Grade, and $w_{i}$ (for i=1,2,3,4,5) are the weights assigned to each ESS level.

By applying the equal weighting method, we assign $w_{1}=w_{2}=w_{3}=w_{4}=w_{5}$. The grades (SPG,AUG,SAG,SSG,AKG) express the fulfillment of the indicators at each level, ranging from 0 (no contribution to energy security) to 1 (maximum contribution to energy security).

Based on the Eurostat publications of EU countries’ energy flows,^42^ we will apply the definition of flows and formulate the energy system balance, which serves as the fundamental equation for deriving our metrics:

(Equation 2) $$I\left(t\right)+P\left(t\right)=D\left(t\right)+E\left(t\right)+L\left(t\right)+S_{b}\left(t\right)-S_{d}\left(t\right),\text{for}\;\text{all}\;t\in T\text{,}$$

where I(t) is the import of all energy flows, P(t) is the domestic production of all energy flows, D(t) is the demand of all energy flows, E(t) is the export of all energy flows, L(t) is the loss of all energy flows, $S_{b}\left(t\right)$ is the stock build of all energy flows, $S_{d}\left(t\right)$ is the stock drawn of all energy flows, and $T=\left[t_{0},t_{1}\right]$, for some start time $t_{0}$ and end time $t_{1}$.

*T* is the analysis period, which may typically be one year, but it can be any time range of interest. Furthermore, each energy carrier’s flows must be expressed in positive values, $F_{i}\left(t\right)\geq 0$ for all t∈T. Demand *D* at the very basic level of ESS (Self-Production) relates to essential demand. At all other levels, *D* includes both essential and non-essential demand.

Figures 3 and 4 present Sankey diagrams for Switzerland, constructed in accordance with Eurostat principles. The 2022 values are taken from the Gesamtenergiestatistik 2022 of the Swiss Federal Office of Energy (SFOE),^25^ whereas the 2050 data are based on the “integriert-offensiv” scenario of the VSE Energiezukunft 2050.^26^

Before presenting the metrics, we introduce the set of energy carriers whose summation over the analyzed period is non-zero. This set will be used to compute various metrics below. We denote this set as M(·,·) and define it as

(Equation 3) $$M\left(F,T\right)=\left\{i\;|\int_{T}F_{i}\left(t\right)\;dt>0,i\in \left\{1,\ldots ,n\right\}\right\}\text{,}$$

where *F* is an energy flow, such as the produced energy *P*, the demand *D*, or the imported energy *I*, depending on the analyzed level.

Self-Production Grade (SPG) refers to the ability of a country, region, or entity to generate its own energy by utilizing domestic Stocks, expressed as $\int_{T}P_{i}\left(t\right)\;dt$. This foundational level emphasizes locally installed energy production capacity to meet at least essential energy needs. The key technical indicator here is the production of own energy relative to the demand over the analysis horizon *T*, putting domestic energy generation in the context of the total energy demand $\int_{T}D\left(t\right)\;dt$. The SPG is evaluated for those carriers only whose flow is non-zero over the considered horizon.

With M(·,·) as defined in Equation 3, the SPG is measured as

(Equation 4) $$SPG=d\left(P,M\left(P,T\right)\right)\;\frac{\sum_{i\in M\left(P,T\right)}\left(\int_{T}\left(P_{i}\left(t\right)-L_{i}\left(t\right)\right)\;dt+Sto_{i}\left(t_{1}\right)-Sto_{i}\left(t_{0}\right)\right)}{\sum_{i}\int_{T}D_{i}\left(t\right)\;dt}\text{,}$$

where d(·,·) is the normalized Shannon-Wiener Diversity Index,^43^ which is evaluated for *P* and the set M(P,T), addressing the diversity aspect introduced in Section cross-cutting measures to enhance energy supply security as

(Equation 5) $$d\left(P,M\left(P,T\right)\right)=-\frac{\sum_{i\in M\left(P,T\right)}\Phi_{i}\left(P,M\left(P,T\right)\right)\;\ln\left(\Phi_{i}\left(P,M\left(P,T\right)\right)\right)}{\ln\left(\left|M\left(P,T\right)\right|\right)}\text{,}$$

(Equation 6) $$\Phi_{i}\left(P,M\left(P,T\right)\right)=\frac{\int_{T}P_{i}\left(t\right)\;dt}{\sum_{i\in M\left(P,T\right)}\int_{T}P_{i}\left(t\right)\;dt},\text{for}\;i\in M\left(P,T\right)\text{,}$$

where $P_{i}$ is the domestic production of energy flow *i*, $D_{i}$ is the demand for energy flow *i*, $L_{i}$ is the loss of energy flow *i*, $Sto_{i}\left(t_{1}\right)$ and $Sto_{i}\left(t_{0}\right)$ are the stocks of energy flow *i* at the end and start of the analyzed period, and |M| denotes the cardinality of the set M.

Autonomy Grade (AUG) refers to the degree of control or independence of a nation over its energy flows. Since domestically produced flows are fully controlled by the nation’s stakeholders (e.g., private sector, government entities, and regulatory bodies which shape infrastructure development, market operations, and policy frameworks), the analysis of autonomy focuses on weighing imported flows *I* and their stock Sto reserves in relation to the demand *D*. The AUG can be improved by diversifying import routes d(I,M(I,T)), addressed in Section cross-cutting measures to enhance energy supply security, establishing robust supplier contracts, expressed by the autonomy factor $a_{i}$, and increasing the normalized reserve capacity of the imported energy carriers ${\left\{\vartheta_{i}\right\}}_{i\in M_{\cap }}$.

All three parameters are derived from the work of Le Coq and Paltseva,^35^ who introduced the Risky External Energy Supply (REES) indicator. The REES framework incorporates factors such as import fuel shares, import fungibility, political risk, the distance between supplier and consumer countries, and overall import dependency. By applying this approach, it is possible to quantify $a_{i}$ through a sophisticated method, reducing the reliance on expert judgment in estimating these values.

With M(I,T) and M(D,T) defined as in Equation 3, and $M_{\cap }=M\left(I,T\right)\cap M\left(D,T\right)$ being the intersection of the two sets, the AUG is calculated by taking the weighted average of all import flows contributing to cover any demand as

(Equation 7) $$AUG=\left(1-\phi \right)+a^{\prime }\;d\left(I,M_{\cap }\right)\;\phi \text{,}$$

(Equation 8) $$\phi =\frac{\sum_{j}\int_{T}I_{j}\left(t\right)\;dt}{\sum_{j}\int_{T}\left(D_{j}\left(t\right)+L_{j}\left(t\right)\right)\;dt},$$

(Equation 9) $$a^{\prime }=\sum_{i\in M_{\cap }}\left(\left(a_{i}+\left(1-a_{i}\right)\;\vartheta_{i}\right)\;\frac{\int_{T}I_{i}\left(t\right)\;dt}{\sum_{j\in M_{\cap }}\int_{T}I_{j}\left(t\right)\;dt\;}\right)\text{,}$$

(Equation 10) $$\vartheta_{i}=\min\left(\frac{Sto_{i}\left(t_{1}\right)}{n_{i}\;\int_{T}\left(D_{i}\left(t\right)+L_{i}\left(t\right)\right)\;dt},1\right),\text{for}\;i\in M_{\cap }\text{,}$$

where d(·,·) is as in Equations 5 and 6, but here evaluated for the imported energy flow *I* if contributing to a demand, $a_{i}$ is the autonomy factor of import flow *i.* Note that the analyst must assess the security of each import $I_{i}$ by either calculating $a_{i}$ using the REES framework or estimating $a_{i}$ in a range from 0 (insecure import) to 1 (fully secure import that remains available even during unexpected events). Furthermore, $n_{i}$ is the reserve factor relative to the demand and loss for the energy flow *i*, and $Sto_{i}\left(t_{1}\right)$ is the reserve capacity of the energy flow *i* at the end of the analysis horizon. In Equation 10, the numerator is the energy content of the storage of the respective energy carrier *i*, the integral in the denominator normalizes this storage by the sum of the demand and losses, and the factor $n_{i}>0$ is a strategic factor. For example, suppose the horizon *T* is one year. Then, if an energy strategy dictates that one should have reserves that last two years, one would set $n_{i}=2$. A less secure supply strategy that only requires storage covering half a year of demand and losses would set $n_{i}=0.5$.

System Adequacy Grade (SAG) measures the reliability of energy flows to meet demand at any instant. While Self-Production and Autonomy are evaluated through an aggregated assessment of energy flows, System Adequacy uses a time-dependent indicator that measures whether energy flows are sufficient to meet demands at all times. This includes both domestic production and imports. Additionally, System Adequacy encompasses operational flexibility, which is represented by the availability of dispatchable capabilities, reserves, and network capacities *c* that can be deployed during unexpected events. This ensures that the energy system can adapt to fluctuations and maintain a stable supply under varying conditions, reaching a *c* of 1. However, if the *i*-th energy carrier is, e.g., non-dispatchable and solely reliant, for example, on photovoltaic production, then $c_{i}=0$. System Adequacy is defined as

(Equation 11) $$SAG=\sum_{i}\left(\frac{c_{i}}{T}\int_{T}f_{i}\left(t\right)\frac{D_{i}\left(t\right)}{\sum_{j}D_{j}\left(t\right)}\;dt\right)\text{,}$$

with $f_{i}\left(\cdot \right)$ being the indicator function

(Equation 12) $$f_{i}\left(t\right)=\left\{ \begin{array}{cc} 1, & \text{if}\;P_{i}\left(t\right)+I_{i}\left(t\right)+E_{i}\left(t\right)+S_{d,i}\left(t\right)-S_{b,i}\left(t\right)-L_{i}\left(t\right)\geq D_{i}\left(t\right),\\ 0, & \text{otherwise}, \end{array}\right.$$

where $c_{i}$ is the capacity factor of energy flow *i*, with $c_{i}\in \left[0,1\right]$ for all i∈{1,…,n}, $P_{i}$ is the domestic production of energy flow *i*, $E_{i}$ is the export of energy flow *i*, whereas export requirements must be met to achieve System Adequacy, ensuring that exports are reliably maintained, $I_{i}$ is the import of energy flow *i*, $D_{i}$ is the demand for energy flow *i*, $L_{i}$ is the loss of energy flow *i*, $S_{b,i}$ is the storage build of energy flow *i*, and $S_{d,i}$ is the storage drawn of energy flow *i*.

Self-Sufficiency Grade (SSG) measures the ability to operate independently from imported flows, relying solely on its own production and storage capacities at given times. We define Self-Sufficiency as

(Equation 13) $$SSG=\sum_{i}\left(\frac{1}{T}\int_{T}g_{i}\left(t\right)\frac{D_{i}\left(t\right)}{\sum_{j}D_{j}\left(t\right)}\;dt\right)\text{,}$$

with $g_{i}\left(\cdot \right)$ being the indicator function

(Equation 14) $$g_{i}\left(t\right)=\left\{ \begin{array}{cc} 1, & \text{if}\;P_{i}\left(t\right)+S_{d,i}\left(t\right)-S_{b,i}\left(t\right)-E_{i}\left(t\right)-L_{i}\left(t\right)\geq D_{i}\left(t\right),\\ 0, & \text{otherwise}, \end{array}\right.$$

where $P_{i}$ is the domestic production of energy flow *i*, $D_{i}$ is the demand of energy flow *i*, $L_{i}$ is the loss of energy flow *i*, $E_{i}$ is the export of energy flow *i*, $S_{b,i}$ is the storage built of energy flow *i*, and $S_{d,i}$ is the storage drawn of energy flow *i*.

Autarky Grade (AKG) represents the highest level of energy independence, where a country or entity can operate entirely independently of imported flows at all times. It implies a state of complete self-reliance and isolation from global energy dynamics, which, while offering the highest form of ESS, may not always be practical or desirable due to economic, technological, or environmental considerations. We describe a system as autarkic if it is self-sufficient and independent from external resources:

(Equation 15) AKG=SSG=1.

The metrics are interrelated, as each one captures a distinct aspect of the analyzed energy system. While achieving a high score on any given tier highlights an essential facet of ESS, a comprehensive assessment requires evaluating all five dimensions. These dimensions probe different aspects of system behavior—for instance, balancing annual energy demand (Autonomy) versus ensuring hourly supply-demand matching (System Adequacy).

By examining how various flows interact within an energy system, planners, policymakers, and researchers can recognize strategic opportunities to enhance both the technical and non-technical dimensions of ESS across all tiers. This structured, hierarchical approach not only offers a comprehensive quantitative picture of a system’s current level of ESS but also guides informed strategic planning for the energy system transformation.
